# RNA editing affects cis‐regulatory elements and predicts adverse cancer survival

**DOI:** 10.1002/cam4.4146

**Published:** 2021-07-28

**Authors:** Yuan‐Ming Wu, Yan Guo, Hui Yu, Tao Guo

**Affiliations:** ^1^ School of Basic Medical Sciences Guizhou Medical University Guiyang China; ^2^ Stem Cell and Tissue Engineering Research Center Guizhou Medical University Guizhou China; ^3^ Comprehensive Cancer Center University of New Mexico Albuquerque NM USA; ^4^ Guizhou Provincial People’s Hospital Guiyang China

**Keywords:** cancer prognosis, overall survival, RNA editing

## Abstract

**Background:**

RNA editing exerts critical impacts on numerous biological processes and thus are implicated in crucial human phenotypes, including tumorigenesis and prognosis. While previous studies have analyzed aggregate RNA editing activity at the sample level and associated it with overall cancer survival, there is not yet a large‐scale disease‐specific survival study to examine genome‐wide RNA editing sites’ prognostic value taking into account the host gene expression and clinical variables.

**Methods:**

In this study, we solved comprehensive Cox proportional models of disease‐specific survival on individual RNA‐editing sites plus host gene expression and critical demographic covariates. This allowed us to interrogate the prognostic value of a large number of RNA‐editing sites at single‐nucleotide resolution.

**Results:**

As a result, we identified 402 gene‐proximal RNA‐editing sites that generally predict adverse cancer survival. For example, an RNA‐editing site residing in *ZNF264* indicates poor survival of uterine corpus endometrial carcinoma, with a hazard ratio of 2.13 and an adjusted *p*‐value of 4.07 × 10^−7^. Some of these prognostic RNA‐editing sites mediate the binding of RNA binding proteins and microRNAs, thus propagating their impacts to extensive regulatory targets.

**Conclusions:**

In conclusion, RNA editing affects cis‐regulatory elements and predicts adverse cancer survival.

## Abstract 2

RNA editing events have been shown to be associated with cancer, and drug sensitivity. We show that for the first time, RNA editing level is associated poor cancer prognosis after adjusting for host gene expression and other phenotypical variables.

## 1 INTRODUCTION

In humans, RNA editing causes nucleotide substitutions in RNA as compared to the corresponding DNA sequence. Of all 12 possible types of nucleotide substitutions, adenine‐to‐inosine (A‐to‐I) editing prevails with a clearly defined biological mechanism. In more detail, A‐to‐I RNA‐editing is mediated by adenosine deaminase acting on RNA (ADAR)^1^ and it accounts for over 95% of all RNA‐editing events.^2^ Given the overwhelming predominance of A‐to‐I RNA‐editing and doubts of non‐canonical editing events,^3^ most studies tend to focus on the type of A‐to‐I RNA‐editing only. While early studies regard RNA‐editing as a binary event, that is, judging its presence or absence qualitatively, recent studies begin to quantify RNA‐editing level – a quantitative attribute that can be calculated as, for example, the ratio between edited reads (reads supporting alternative allele) and total reads. Chigaev et al. have taken this quantitative perspective to observe elevated RNA‐editing levels in tumors compared to paired normal samples in 11 cancer types.^3^

RNA editing has the potential to impact cellular processes and affect diseases such as cancers. For example, Peng et al. demonstrated experimentally that nonsynonymous A‐to‐I RNA‐editing can result in alternative protein sequences,^4^ and these changes may subsequently affect anti‐cancer drug sensitivity.^5^ As a concrete example, RNA editing in *AZIN1* in hepatocellular carcinoma can trigger more aggressive tumors by causing higher cell proliferation through the neutralization of antizyme‐mediated degradation of ornithine decarboxylase and cyclin D1.^6^ In another study, RNA editing was shown to cause important gain or loss of binding sequences of RNA binding proteins (RBPs), microRNA (miRNA) seeds, and miRNA‐matching 3’‐UTRs, thus leading to reprogrammed regulatory cascades.^7^

The research field witnessed sporadic studies of RNA‐editing’s cancer prognostic value,^8^, ^9^ and both increased^10^, ^11^ and decreased^12^, ^13^ RNA‐editing levels have been noted in various tumors. Two recent review articles^14^, ^15^ have put together around twenty genes that bear RNA‐editing sites (RESs) of prognostic marker value. Paz‐Yaacov et al.^16^ summarized A‐to‐I RNA‐editing events in each tumor sample as RNA‐editing index, and, in such a sample‐aggregated perspective, proposed that increased editing activity is associated with poor prognosis. In the current work, we followed the same direction to investigate RNA‐editing’s cancer prognostic value; our innovation lies foremost in that we set individual RESs as the research units so that we managed to analyze transcriptome‐wide RNA‐editing events at a single‐nucleotide resolution. Other than that, we devised the study along the following three aspects of novelty. First, the previous studies did not account for correlation between gene expression and RNA editing level, which posed a risk for identifying false positive RESs that were simply proxies of their host genes’ expression. In our study, we first showed that RNA editing level is often strongly correlated to host gene expression, and we went on to let this important awareness guide our survival analyses. Second, we adjusted for clinical variables (age, sex, stage) in our Cox model, which was not done in most previous studies. Last and most importantly, we analyzed disease‐specific survival, which was advocated as a more accurate outcome variable than overall survival.^17^

We collected 99,071 distinct A‐to‐I RNA‐editing sites originating from patient samples of various cancers and explored their prognostic value with proper adjustment of crucial covariates. First, we showed that RNA‐editing level is positively associated with the expression of the host gene. This finding validated our intuitive hypothesis and justified our deliberate adjustment of gene expression in the next‐step survival analysis. Several thoughtful survival models were applied to nearly a hundred thousand A‐to‐I RNA‐editing sites, and, after multiple test adjustments, we fetched 402 gene‐proximal RNA‐editing sites that were significantly associated with survival, mostly in the adverse direction. Some of these prognostic RNA‐editing sites disrupted regulation cascades by modifying RBP binding sequences or miRNA‐matching 3′‐UTRs.

## 2 METHODS

### 2.1 Data acquisition and annotation

Technically, RNA‐editing events usually are detected with RNA‐Seq data. The RNA‐editing‐occurring genomic position is recognized as an RNA‐editing site (RES). A‐to‐I RNA‐editing events originating from RNA‐Seq data of The Cancer Genome Atlas (TCGA) were obtained as supplementary data from previous studies.^3^, ^5^ These data were detected in 5672 patient subjects of 17 cancer types, comprising 99,071 distinct RESs. A catalog of these RNA‐editing events, including the related cancer type information, is provided in Table S1. TCGA uses a stable acronym series to code the long yet accurate cancer names, and the acronyms of the 17 cancer types (BLCA, BRCA, CESC, CRC, GBM, HNSC, KICH, KIRC, KIRP, LGG, LIHC, LUAD, LUSC, PRAD, STAD, THCA, and UCEC) are explained in Table S1.

We leveraged ANNOVAR^18^ to annotate genomic regions for RESs. Based on the dissection of the human reference genome, a RES was allocated to one of nine possible genomic regions: exonic, intronic, 5′‐UTR, 3′‐UTR, ncRNA, upstream, downstream, intergenic, and splicing. Whenever a RES was allocated to the intergenic region, it was considered a gene‐distal RES; RESs allocated to any other genomic regions, including gene upstream and gene downstream regions (within 1000 bp of transcription start site or transcription end site), were collectively termed gene‐proximal RESs. For each gene‐proximal RES, a host gene was identified as the nearest gene whose gene body hosted or approximated the RES in question. The whole set of 99,071 RESs was thus allocated to 6254 distinct host genes. Of note, all chromosome coordinate positions specified in this manuscript were based on the human GRCh37 reference genome.

From Genomics Data Commons, we downloaded TCGA RNA‐seq gene expression data in the format of fragment per kilobase million; from the same source, we obtained clinical covariate information for the TCGA patient cohort, including age, gender, and cancer stage. From TCGA Pan‐Cancer Clinical Data Resource,^17^ we acquired disease‐specific survival information.

### 2.2 Term definition

By definition, an RNA‐editing event must involve a RES, and one same RES may be involved in different RNA‐editing events where different subjects or patient cohorts were concerned. Therefore, we defined RNA‐editing level for a RES in one subject, RNA‐editing frequency for a RES in one cohort (typically a cancer type), and RNA‐editing density for a gene that hosts multiple RESs.

(1) $$L_{i}^{C}\left(j\right)=\frac{R_{i,j}^{+}}{\left(R_{i,j}^{+}+R_{i,j}^{‐}\right)}$$

(2) $$F_{i}^{C}=\frac{\left\{Sub_{j}|L_{i}^{C}\left(j\right)\rangle 0\right\}}{\left\{Cohort\left(C\right)\right\}}$$

(3) $$D\left(g\right)=\frac{\sum_{i\in G}I^{*}\left(\sum_{C}\sum_{j}I^{*}\left(L_{i}^{C}\left(j\right)>0\right)>0\right)}{\left|\left|G\right|\right|}$$

As indicated in Equation (1), RNA‐editing level (*L*) of RES *i* in subject *j* (of cohort *C*) was expressed as the ratio of edited reads $\left(R_{\left(i,j\right)}^{+}\right)$ over total reads $\left(R_{\left(i,j\right)}^{+}\;\mathrm{and}\;R_{\left(i,j\right)}^{‐}\right)$. Edited reads support alternative, non‐reference allele at the particular genomic position. RNA‐editing level took value over interval [0,1].

As indicated in Equation (2), RNA‐editing frequency (*F*) of RES *i* in a cancer type‐specific cohort (denoted as *C)* was expressed as the ratio of subjects showing RNA‐editing at RES *i* over total cohort size. RNA‐editing frequency took value over interval [0,1]. In all cancer types and all genomic regions, we did observe RESs with a full frequency (1).

As indicated in Equation (3), RNA‐editing density (*D*) of gene *g* was expressed as the rate of RESs within their host gene body, expressed as the number of RESs with non‐zero RNA‐editing level (in any subject of any cohort) divided by the length of the host gene. Here, *G* designated the gene body of the host gene *g*, interpretated as a set of continuous sites, and therefore *||G||* designates the gene length in nt; *i* was the location identifier of an RES, with i∈G meaning gene *g* was the host gene of RES *i*. I*(x) designated the indicator function where a logic expression *x* was evaluated and either 1 or 0 was returned. RNA‐editing density took value over interval (0,1).

### 2.3 Statistical analysis

We conceptualized two models of dependence of RES level (*L*) on host gene expression (*E*), one continuous (Equation 4) and the other binary (Equation 5). Continuous RES level was calculated as explained above (Equation 1) and was modified slightly to go into the left side of the equations: in the continuous model (Equation 4), original RES level was standardized to a new continuous variable $\left(L^{*}\right)$ that followed the standard normal distribution; in the binary model (Equation 5), it was dichotomized to binary values of 0’s and 1’s$\left(L^{\prime }\right)$. Logistic function was denoted as *logit(x)*. Gene expression value was processed from initial fragment per kilobase million values with a state‐of‐the‐art protocol including logarithm and normalization. Linear regression and logistic regression were conducted to solve the coefficient (*b*) in the models.

(4) $$L^{*}=a+b\cdot E$$

(5) $$logit\left(Prob(L^{\prime }=1\right))=a+b\cdot E$$

(6) $$\frac{\lambda \left(t\right)}{\lambda_{0}\left(t\right)}=\exp\left(c_{l}\cdot L\right)$$

(7) $$\frac{\lambda \left(t\right)}{\lambda_{0}\left(t\right)}=\exp\left(c_{l}\cdot L+c_{e}\cdot E\right)$$

(8) $$\frac{\lambda \left(t\right)}{\lambda_{0}\left(t\right)}=\exp\left(c_{l}\cdot L+c_{e}\cdot E+\left(c_{\mathrm{stg}}\cdot Stg+c_{\mathrm{age}}\cdot Age+c_{\mathrm{sex}}\cdot Sex\right)\right)$$

(9) $$\frac{\lambda \left(t\right)}{\lambda_{0}\left(t\right)}=\exp\left(c_{e}\cdot E+\left(c_{\mathrm{stg}}\cdot Stg+c_{\mathrm{age}}\cdot Age+c_{\mathrm{sex}}\cdot Sex\right)\right)$$

(10) $$\frac{\lambda \left(t\right)}{\lambda_{0}\left(t\right)}=\exp\left(c_{l}\cdot L+\left(c_{\mathrm{stg}}\cdot Stg+c_{\mathrm{age}}\cdot Age+c_{\mathrm{sex}}\cdot Sex\right)\right)$$

We modeled patient disease‐specific survival with three gradually more comprehensive Cox proportional hazard models (Equations 6, 7, 8). In the most primitive model (Equation 6), death hazard $\left(\frac{\lambda \left(t\right)}{\lambda_{0}\left(t\right)}\right)$ was explained by RES level (*L*) only; in an intermediate model (Equation 7), death hazard was explained by RES level plus host gene expression (*E*); in the most comprehensive model (Equation 8), RNA‐editing level contribution to death hazard was adjusted for host gene expression as well as multiple clinical variables, including stage (*Stg*), age (*Age*), and sex (*Sex*). These Cox models were resolved within each cancer type separately, where data of all subjects relevant to one‐specific RES were utilized to resolve the coefficients $\left(c_{l},c_{e},c_{\mathrm{stg}},c_{\mathrm{age}},c_{\mathrm{sex}}\right)$. Of note, for gender‐specific cancer types (BRCA, PRAD, and OV), the sex term $\left(c_{\mathrm{sex}}\cdot Sex\right)$ was omitted in the most comprehensive model (Equation 8).

To specifically pinpoint the contribution to death hazard from RNA‐editing level, we constructed a variant to the most comprehensive model so that the RES term was left out (Equation 9). The two Cox models, with and without the RES term (Equations 8 and 9), were compared, and the concordance index (C‐index)^19^ was calculated to assess the sole contribution from the RES’ level to patient's death hazard. The last Cox model as expressed in Equation (10) was applied on gene‐distal RESs where no host gene was associated with a RES.

All statistical analyses were conducted in R environment (v4.0.2). Because RES‐gene dependence analysis and survival analysis were conducted for each individual RES repeatedly, multiple‐test correction was applied to the *p*‐values of bulk RESs with the Benjamini–Hochberg method, and adjusted *p*‐value <0.05 was considered statistically significant. R packages survival and survminer were utilized to perform Cox regression and render Kaplan‐Meier curves.

### 2.4 Analysis of RNA‐editing‐associated binding sequence

When RNA‐editing takes place in cis‐regulatory elements, regulation of gene expression may be affected and the impact of a RES may be propagated to a large number of regulatory targets.^7^ We leveraged Somatic Binding Sequence Analyzer^20^ to identify RES‐affected cis‐regulatory elements. Technically, we screened three classes of cis‐regulatory elements, namely RBP binding sequences, miRNA seed sequences, and miRNA‐matching 3′‐UTR sequences. RBP binding sequences numbered 3524 and were downloaded from four databases: ATtRACT,^21^ ORNAment,^22^ RBPDB,^23^ and RBPmap.^24^ MiRNA seed sequences numbered 2,879 and were downloaded from mirBase.^25^ MiRNA‐matching 3′‐UTR sequences numbered 2,055,403 and were downloaded from starBase v2.0.^26^ Circos plot^27^ was used to manifest a genome‐wide view of RES‐affected cis‐regulatory elements.

### 2.5 Functional characterization of RNA‐editing sites

A set of 379 cellular pathways,^28^ each of 5–236 genes, were merged from PID,^29^ PANTHER,^30^ and INOH^31^ and were used to annotate function themes of RES’ host genes. The same set of pathways were adopted in previous studies on chronic kidney disease^32^ and pan‐cancer survival markers.^33^ Specifically, for each cancer type, we identified the host genes of prognostic RESs (output of Equation 8) and conducted hypergeometric test against each pathway. Since there was a multiple‐test issue here, the Benjamini–Hochberg method was again utilized to adjust the hypergeometric *p*‐values. Adjusted *p*‐value <0.05 was considered statistically significant.

A series of methods are available to assess the functional impact resulting from a variation at a particular genomic position. These methods are generally based on multiple sequence alignment within a protein family, presuming that positions with a low conservation rate are likely to tolerate a mutation while positions with a high conversion rate are likely to be intolerant to a mutation. In light of such a evolutionary perspective, editing impact was predicted for each RES in our final report set, using eight algorithms: SIFT,^34^ Polyphen2 (including both HDIV and HVAR),^35^ LRT,^36^ FATHMM,^37^ CADD,^38^ VEST3,^39^ and MetaSVM.^40^ The scores out of distinct algorithms were normalized to a common scale between 0 to 1, where a higher value signified a stronger impact.

## 3 RESULTS

### 3.1 RNA‐editing level is highest in splicing regions

An overall description of the nearly one hundred thousand RESs, including information on TCGA cancer types, was provided in Table S1. Of the initial 99,071 RESs, 95.8% (94,957) were located in Alu segments. We compared the genomic regions of RESs in terms of raw number, level, and frequency of RESs. Separate analyses in individual cancer types showed consistent patterns, so here we presented consensus results that conglomerated all cancer types.

**FIGURE 1:**
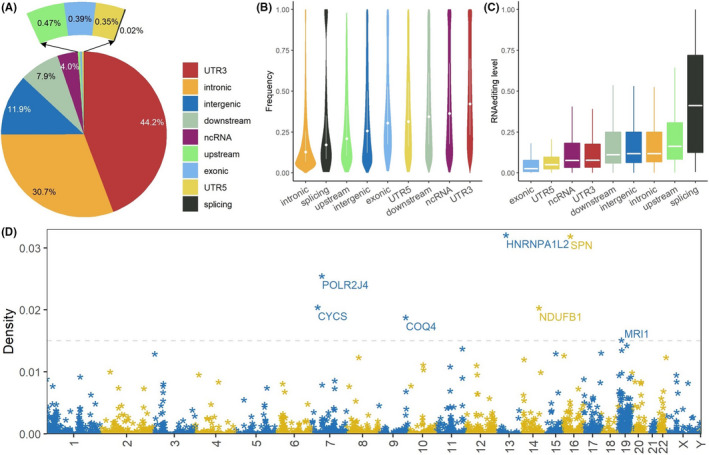
Overall description of RNA‐editing events in 17 cancer types. (A) RNA‐editing sites by genomic regions. (B) RNA‐editing frequency by genomic regions. (C) RNA‐editing level by genomic regions. (D) Manhattan plot for RNA‐editing density. Density is assessed for gene units (Equation 3). Marked genes are cancer‐relevant genes with the highest RNA editing density

Considering the raw number of RESs, 3′‐UTR and intronic region are the most noteworthy genomic regions, as 44.2% of the 99,071 RESs occurred in 3′‐UTR regions followed by 30.7% in introns (Figure 1A). The heavy presence of RNA‐editing in 3′‐UTR and intronic regions is consistent with the previous report.^41^ In such non‐coding regions, Alu elements and other long interspersed nuclear elements are over‐represented, which are conducive to form imperfect doublestrand motifs recognized by ADAR. Around 4.0% RNA‐editing falls into the non‐coding RNA regions. More interestingly, exonic RNA‐editing occupied 0.39% of all RESs. Splicing junction had the lowest RESs at 0.02% occupancy.

In light of RNA‐editing frequency (Equation 2), 3′‐UTR stands out among all nine genomic regions, with a median frequency of 0.42 (Figure 1B). Surprisingly, intronic region shows the least frequency, with a median of 0.128. In light of RNA‐editing level (Equation 1), a completely different picture is revealed – splicing region shows the highest level, with a median of 0.411 (Figure 1C). On the contrary, exonic region shows the lowest RNA‐editing level, with a median of 0.26. Exons are more evolutionarily conserved, thus it makes sense to display both less number and less RNA‐editing level. Splicing junctions have the lowest number of RNA‐editing yet the highest RNA‐editing level. This may indicate the critical involvement of RNA editing in the transcriptional regulatory system.

Lastly, we examined RNA‐editing density (Equation 3) for genes located to all 24 chromosomes (Figure 1D). Several hyper‐RNA‐edited genes were identified, including the three top‐ranking ones: *HNRNPA1L2*, *SPN*, and *POLR2J4*. *HNRNPA1L2* is known to fuse with *SUGT1* in cervical^42^ and bladder^43^ cancers. *SPN*, a regulatory subunit of *PP1A*, is a known tumor suppressor.^44^ *POLR2J4* is a non‐coding RNA and has recently been shown to be associated with survival in hepatocellular carcinoma by two independent studies.^45^, ^46^ Hosting dense RESs in the gene body may add to the evidence of cancer relevance of these genes.

### 3.2 RNA‐editing level is generally correlated with host gene expression

**FIGURE 2:**
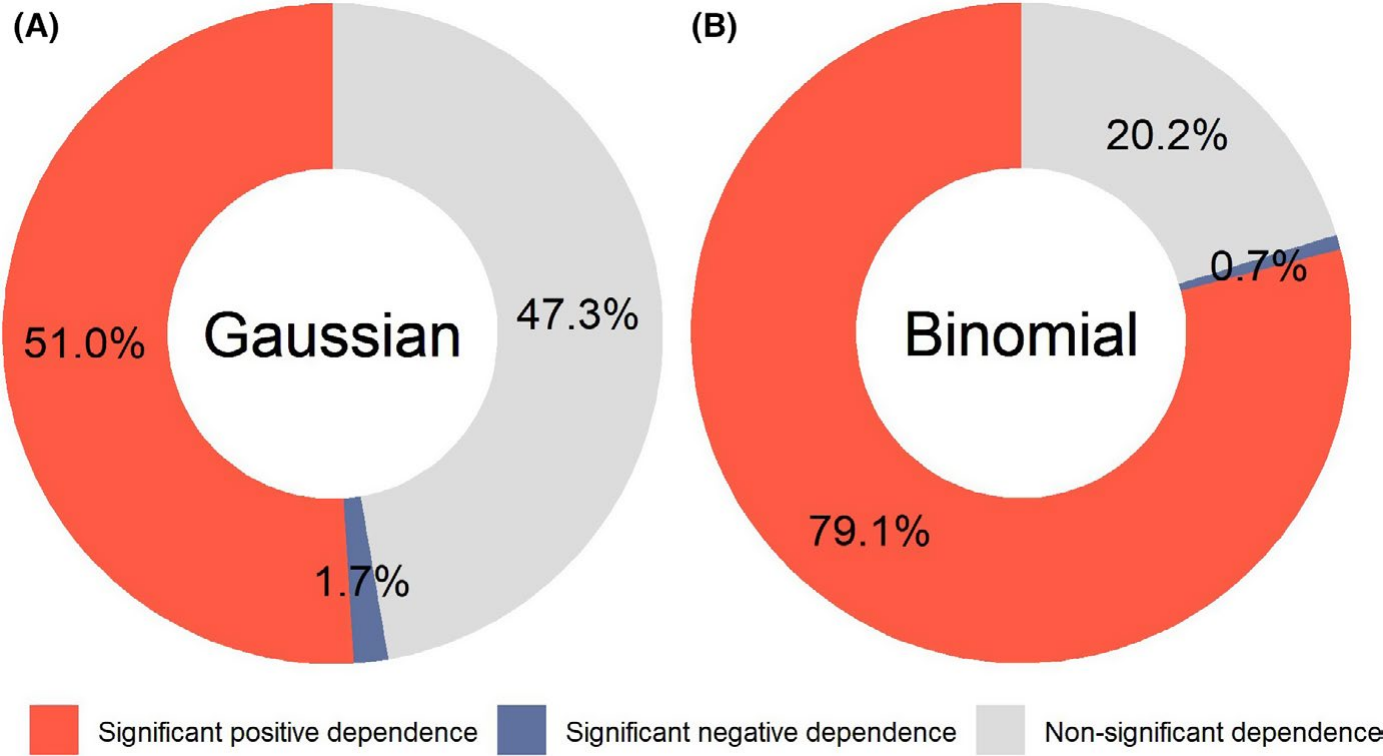
Breakdown of gene‐proximal RNA‐editing sites by the direction of correlation between RNA‐editing level and host gene expression. (A) RNA‐editing level was modeled as a continuous variable and Gaussian (linear) family regression was conducted (Equation 4). (B) RNA‐editing level was modeled as a binary variable and Logistic regression was conducted (Equation 5). In both A and B, positive dependence relations predominate negative ones

A majority of current methods seek to detect RNA editing from RNA‐seq data, where read counts for an allele are inherently correlated with gene expression. As reflected in the definition (Equation 1), RNA‐editing level is built on read counts for the reference allele and the alternative allele. Thus, we hypothesized that the level of a RES is correlated with host gene expression. The presumed dependence of RNA‐editing level on host gene expression was modeled in continuous (Equation 4) and binary (Equation 5) models, respectively. Regression of the continuous model revealed that 51% RESs showed a significant positive correlation with host gene expression and 1.7% showed a negative correlation (Figure 2A). Regression of the binary model revealed that 79.1% RESs showed a significant positive correlation with host gene expression and 0.7% showed a negative correlation (Figure 2B). In both models, a majority of the RES levels were found positively dependent on host gene expression. This finding highlights the necessity of accounting for gene expression in any analysis that revolves around RNA‐editing level, and we did take this precaution into consideration in the following survival analysis where individual RESs were evaluated for their prognostic significance.

### 3.3 RNA‐editing events predict adverse survival in 11 cancers

**FIGURE 3:**
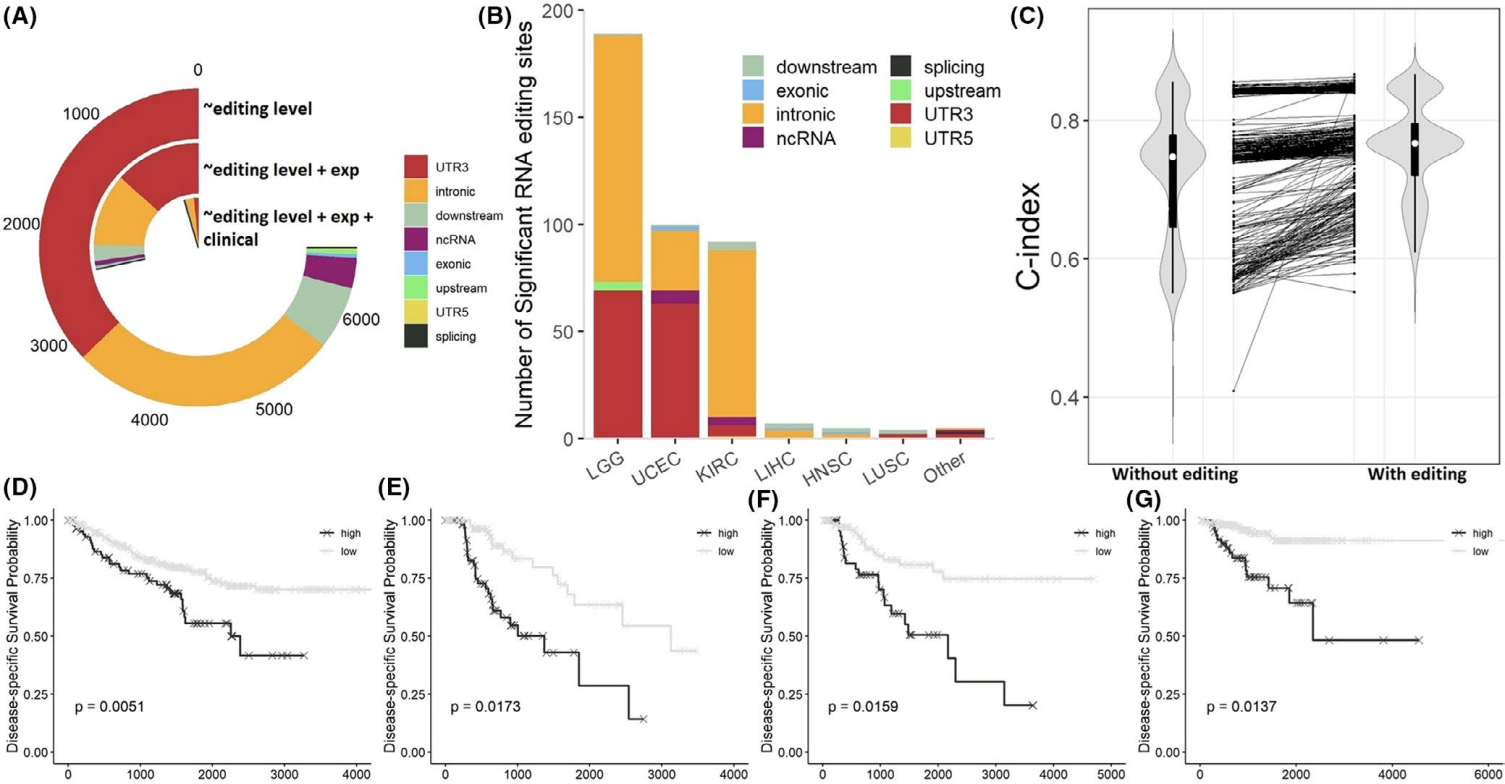
Prognostic value of gene‐proximal RNA‐editing sites. (A) Numbers of significant RNA‐editing sites resulting from three survival models (Equations 6, 7 and 8). The number of significant RNA‐editing sites dropped dramatically when host gene expression was adjusted for in the model. (B) The number of significant RNA‐editing sites by cancer. (C) C‐index values generally increased when RNA‐editing level was incorporated into the survival model (Equation 9 vs. Equation 8). (D‐G) Kaplan‐Meier curves for four prognostic RNA‐editing events residing in *TRAPPC4* (D), *SPC24* (E), *SNORA40* (F), and *DCAF16* (G), respectively. The prognostic effects were manifested in kidney renal clear cell carcinoma (D), liver hepatocellular carcinoma (E), lung squamous cell carcinoma (F), and uterine corpus endometrial carcinoma (G), respectively

For gene‐proximal RESs, we modeled patients’ disease‐specific survival with three gradually more comprehensive Cox models: (1) Survival ~RNA‐editing level (Equation 6); (2) Survival ~RNA‐editing level +host gene expression (Equation 7); (3) Survival ~RNA‐editing level +host gene expression +clinical variables (Equation 8). As expected, the number of significant RESs substantially decreased with the incorporation of additional variables (Figure 3A). The most dramatic decrease of significant RES number occurred when host gene expression was incorporated to adjust for RES level contribution. This observation resonated with our concern of the positive correlation between RNA‐editing level and host gene expression, which was highlighted above with numerical experiment results (Figure 2). Without adjusting for host gene expression, a survival model built on the sole variable of RNA‐editing level (Equation 6) could be capturing merely the host gene whose expression dictates the superficial RNA‐editing level. After adjusting for host gene expression and clinical variables (Equation 8), levels of 402 RESs were found significantly associated with disease‐specific survival in 11 cancer types (Table S2). Of these 402 RESs, 94% were located in Alu regions. Their cancer type and genomic region distribution are depicted in Figure 3B. Low‐grade glioma (LGG) had the most significant RESs with 189. Six cancer types, breast invasive carcinoma (BRCA), kidney Chromophobe (KICH), kidney renal papillary cell carcinoma (KIRP), lung adenocarcinoma (LUAD), stomach adenocarcinoma (STAD), and thyroid carcinoma (THCA) did not return any significant RESs as prognostic markers.

The sign of (logged) RES coefficient, or hazard ratio, in the Cox model (Equation 8) instructs on the direction of prognostic effect of an RNA‐editing event. Of the total 402 significant RESs, 368 (91.5%) had their hazard ratio greater than one, indicating that a high editing level results in a poor prognostic outcome. Collectively speaking, RNA editing in our investigated cancer types generally predicts poor survival. The top ten RESs associated with survival ranked by adjusted *p*‐value are available in Table 1.

**TABLE 1:** Ten gene‐proximal RNA‐editing sites with the highest prognosis statistical significance (Equation 8)

RES position	Region	Host gene	HR (95% CI)	Ajusted *p* ^a^	Cancer^b^
chr19:57,725,735	3′‐UTR	ZNF264	2.13 [1.70, 2.66]	4.07 × 10^−7^	UCEC
chr12:113,827,926	Downstream	PLBD2	1.55 [1.33, 1.81]	6.36 × 10^−5^	LGG
chr18:32,829,377	Intronic	ZNF397	1.96 [1.55, 2.49]	6.55 × 10^−5^	LIHC
chr7:128,454,323	Intronic	CCDC136	1.57 [1.35, 1.83]	1.46 × 10^−4^	LGG
chr5:138,620,224	Intronic	MATR3	2.03 [1.55, 2.66]	2.19 × 10^−4^	LIHC
chr1:154,960,151	5′‐UTR	FLAD1	1.32 [1.18, 1.49]	3.35 × 10^−4^	KIRC
chr19:4,653,303	3′‐UTR	TNFAIP8L1	2.00 [1.55, 2.57]	3.77 × 10^−4^	UCEC
chr6:160,101,723	Intronic	SOD2	1.64 [1.37, 1.95]	3.94 × 10^−4^	LGG
chr8:144,672,955	Intronic	EEF1D	1.50 [1.29, 1.74]	4.10 × 10^−4^	LGG

Note

Chromosome position is indexed in GRCh37. RES, RNA‐editing site.

Abbreviation: HR, Hazard ratio. CI, Confidence interval.

^a^ Adjusted *p*‐value from survival analysis (Equation 8).

^b^ Full cancer names are expanded in Table S1.

We also exerted another procedure to validate the prognostic value of these recommended RESs, where we computed C‐index between two alternative models, one with RES term (Equation 8) and the other without (Equation 9). The difference of C‐index values between the two models indicates the incremental goodness of fit brought forth by RNA‐editing level. Of the 402 significant RESs, 396 showed increased C‐index values, proving net increment of goodness of fit for the survival model (Equation 8) attributed unambiguously to the incorporated RES (Figure 3C).

Using Kaplan‐Meier curves, we show visually four examples of RNA‐editing level's association with survival. In the first example, an RES of host gene *TRAPPC4* located at chr11:118,893,191 (chromosome 11, position 118,893,191) was associated with poor survival in kidney renal clear cell carcinoma (KIRC) (adjusted *p* = 0.0051) (Figure 3D). There has not been any previous report on *TRAPPC4* with kidney renal clear cell carcinoma. The second example concerns RES of host gene *SPC24* located at chr19: 11,257,198 in liver hepatocellular carcinoma (LIHC) (adjusted *p* = 0.0173) (Figure 3E). *SPC24* has been considered as a biomarker for liver cancer and is upregulated in LIHC tumors.^47^ The third example RES resides in *SNORA40* at chr11:93,468,111 and demonstrated prognosis significance in lung squamous cell carcinoma (LUSC) (adjust *p* = 0.0159) (Figure 3F). *SNORA40* is a small nucleolar RNA and has been proposed as a biomarker for several cancer types.^48^, ^49^ However, no link has been reported for LUSC. The last example RES occurs in *DCAF*16 at chr4:17,804,740 in uterine corpus endometrial carcinoma (UCEC) (adjust *p* = 0.0137) (Figure 3G). *DCAF*16 is DDB1‐CUL4 associated factor. It has been linked to cancer previously.

For gene‐distal RESs which are located in the intergenic region, since they could not be allocated to a nearby host gene, the disease‐specific survival was modeled with a combination of RES and applicable clinical variables (Equation 10). As a result, 311 significant gene‐distal RESs protruded from this screening (Table S3). Of these 311 RESs, 93.9% were located in Alu regions. The signs of (logged) RES coefficients also indicated a general negative survival association, with 259 (83.3%) RESs having greater than one hazard ratio.

### 3.4 RNA‐editing affects cis‐regulatory elements

**FIGURE 4:**
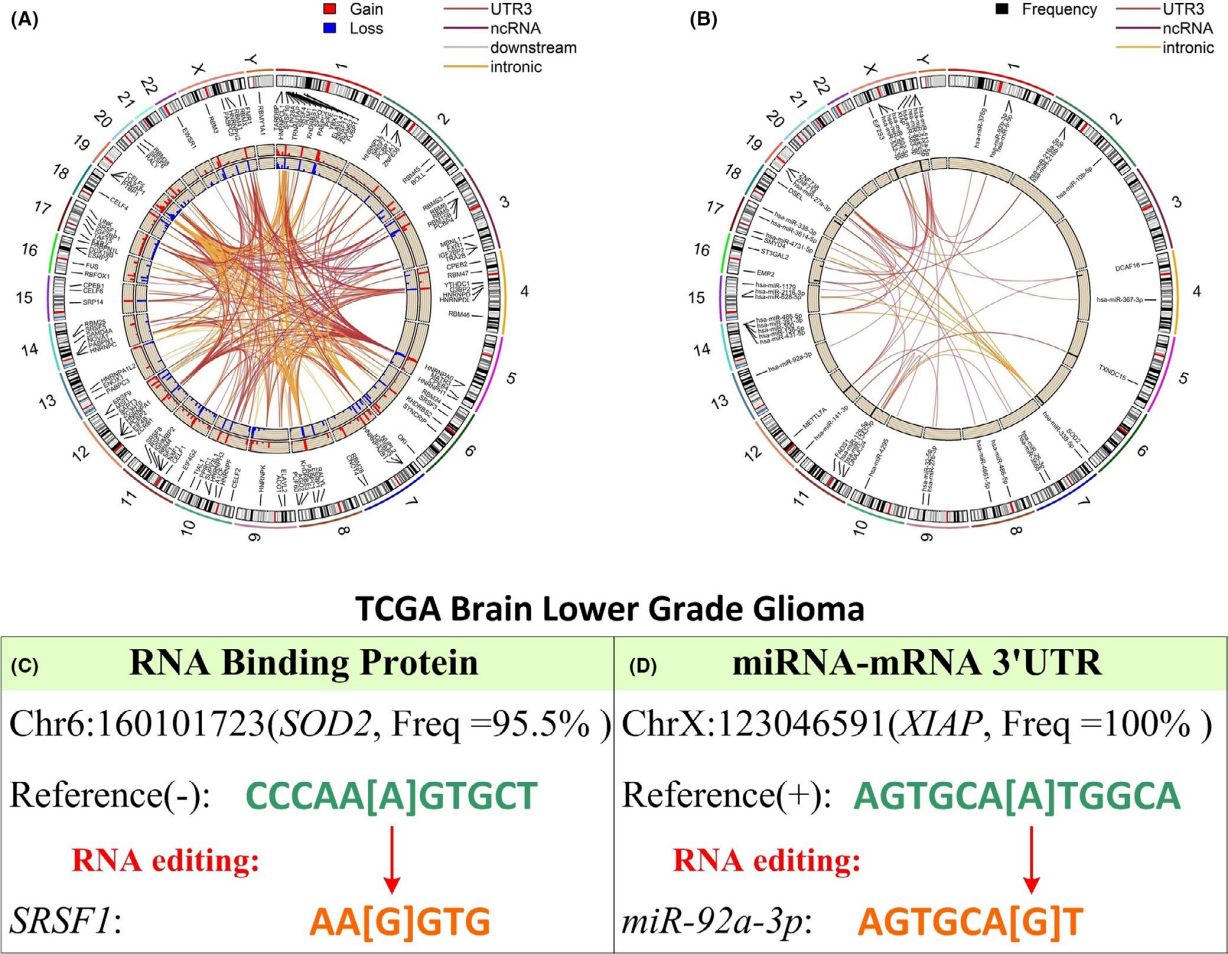
Analysis results of RNA‐editing‐associated binding sequence. (A) Circos plot presenting RNA‐editing‐site‐affected binding sequences for RNA‐binding proteins. RNA‐binding protein names are printed on the plot and can be read when zoomed in. RNA editing caused RBP binding sequence changes are linked by different color lines presenting RNA editing genomic location. The blue bars in the inner circle indicate the loss of RBP binding sequence. The red bars in the second circle denote the gain of the RBP binding sequence. The height of the bars indicates the RNA editing frequency. (B) Circos plot presenting RNA‐editing‐site‐affected miRNA‐matching 3′‐UTR sequences. RNA editing site is linked to the affected miRNA‐matching 3′‐UTRs by different color lines representing RNA editing's genomic region. The black bars indicate the RNA‐editing frequency. (C) An example of gain of RBP binding sequence. RNA‐editing in host gene *SOD2* caused a gain of binding sequence AAGGTG for RBP SRSF1. (D) An example of altered miRNA‐matching 3′‐UTRs binding sequence. RNA‐editing in host gene *XIAP* caused a change in the binding sequence to miRNA miR‐92a‐3p

We performed binding sequences analysis to identify cis‐regulatory elements affected by the 402 prognostic gene‐proximal RESs. Out of 402 RESs, 383 affected cis‐regulatory elements. Precisely, they caused 1177 gains and 1206 losses of RBP binding sequence (Figure 4A, Table 2, and Table S4) and 79 altered miRNA‐matching 3′‐UTRs (Figure 4B, Table 3, and Table S5). We elaborate on two representative examples, one involving an RBP binding sequence (Figure 4C) and the other involving a miRNA‐matching 3′‐UTR sequence (Figure 4D). Firstly, a RES that is located at chr6: 160,101,723 and designated to host gene *SOD2* caused a gain of binding sequence for RBP SRSF1 (Figure 4C). This RNA‐editing event occurred in 95.5% of LGG subjects. Both *SOD2* and *SRSF1* are known for their glioma connections. *SOD2* is a key enzyme with a dual role in tumorigenesis and tumor progression in multiple cancers.^50^ *SOD2* inhibitor treatment was effective to lower cell proliferation in glioma xenograft mouse models.^51^ The RBP SRSF1 promotes glioma tumor via oncogenic splicing of *MY01B* transcript.^52^ Secondly, an RES that is located at chrX:123,046,591 and designated to host gene *XIAP* altered the binding sequence to miRNA *mir*‐*92a*‐*3p* (Figure 4D). *XIAP* is upregulated in glioblastoma,^53^ and *XIAP* inhibitor has been shown effective to treat glioblastoma tumorspheres in vitro.^54^ *XIAP* was identified as a regulation target of *mir*‐*92a*‐*3p*. This RES in question could potentially disrupt the normal regulation between *mir*‐*92a*‐*3p* and *XIAP*, possibly upregulating *XIAP* expression and leading to tumorigenesis.

**TABLE 2:** Ten most prognostic RNA editing sites that caused gains of binding sequences for RNA‐binding protein

RES position	Region	Host gene	HR (95% CI)	Adjusted *p* ^a^	Frequency	Binding Sequence	RBP	Cancer^b^
chr6:160101723	Intronic	*SOD2*	1.64 [1.37, 1.95]	0.00039	95.5%	AAGGTG	HNRNPF	LGG
chr8:99056338	Intronic	*RPL30*	0.63 [0.53, 0.76]	0.00117	78.2%	TTTTTTG	ELAVL1	LGG
chr19: 39384772	Intronic	*SIRT2*	1.53 [1.28, 1.81]	0.00131	51.6%	CGCTCCG	SRSF1	LGG
chr12:51325383	3′‐UTR	*METTL7A*	1.48 [1.26, 1.73]	0.00519	99.6%	AGCACC	NOVA1	LGG
chr18:65176260	3′‐UTR	*DSEL*	1.46 [1.25, 1.72]	0.00613	69.8%	CGGTGG	FUS	LGG
chr11:65180643	3′‐UTR	*FRMD8*	1.82 [1.41, 2.36]	0.00672	67.2%	TGGAGAT	SRSF1	UCEC
chr8:95804969	3′‐UTR	*DPY19L4*	1.49 [1.25, 1.77]	0.00944	99.8%	CCCGGC	SRSF6	LGG
chr18:65174313	3′‐UTR	*DSEL*	1.42 [1.22, 1.66]	0.00916	50.6%	CGGTGG	FUS	LGG
chr5:130537253	3′‐UTR	*LYRM7*	1.47 [1.24, 1.75]	0.01087	82.3%	CTTTTTA	TIAL1	LGG
chrX:24095285	3′‐UTR	*EIF2S3*	0.64 [0.52, 0.78]	0.01087	100.0%	CGAGCGA	ZC3H10	LGG

Note

Chromosome position is indexed in GRCh37. RES, RNA‐editing site.

Abbreviations: HR, Hazard ratio; CI, Confidence interval.

^a^ Adjusted *p*‐value from survival analysis (Equation 8).

^b^ Full cancer names are expanded in Table S1.

**TABLE 3:** Ten prognostic RNA editing sites of the highest statistical significance that altered miRNA‐matching 3′‐UTR sequences

RES position	Region	Host gene	HR (95% CI)	Adjusted *p* ^a^	Frequency	miRNA	Cancer^b^
chr19:57725735	3′‐UTR	*ZNF264*	2.13 [1.70, 2.66]	4.07E−07	7.2%	miR−339‐5p	UCEC
chr18:65174340	3′‐UTR	*DSEL*	1.50 [1.27, 1.76]	3.01E−03	78.4%	miR−1179	LGG
chr19:21302864	3′‐UTR	*ZNF714*	1.55 [1.29, 1.85]	4.90E−03	28.4%	miR−371a−5p	LGG
chr16:70413590	3′‐UTR	*ST3GAL2*	1.70 [1.36, 2.13]	5.19E−03	27.8%	miR−216a−5p	UCEC
chr12:51324467	3′‐UTR	*METTL7A*	1.42 [1.22, 1.64]	6.13E−03	100.0%	miR−3614‐5p	LGG
chrX:24095220	3′‐UTR	*EIF2S3*	0.61 [0.49, 0.75]	7.66E−03	100.0%	miR−371a−5p	LGG
chrX:123046591	3′‐UTR	*XIAP*	1.53 [1.26, 1.86]	1.27E−02	100.0%	miR−32‐5p	LGG
chr4:17804740	3′‐UTR	*DCAF16*	2.04 [1.47, 2.84]	1.37E−02	49.7%	miR−1343‐3p	UCEC
chr6:160101733	Intronic	*SOD2*	1.43 [1.20, 1.71]	1.39E−02	97.7%	miR−338‐3p	LGG
chr5:134236740	3′‐UTR	*TXNDC15*	1.39 [1.19, 1.63]	1.79E−02	100.0%	miR−141‐3p	LGG

Note

Chromosome position is indexed in GRCh37. RES, RNA‐editing site.

Abbreviations: HR, Hazard ratio. CI, Confidence interval.

^a^ Adjusted *p* value from survival analysis (Equation 8).

^b^ Full cancer names are expanded in Table S1.

### 3.5 Functional characterization of our reported RNA‐editing sites

**FIGURE 5:**
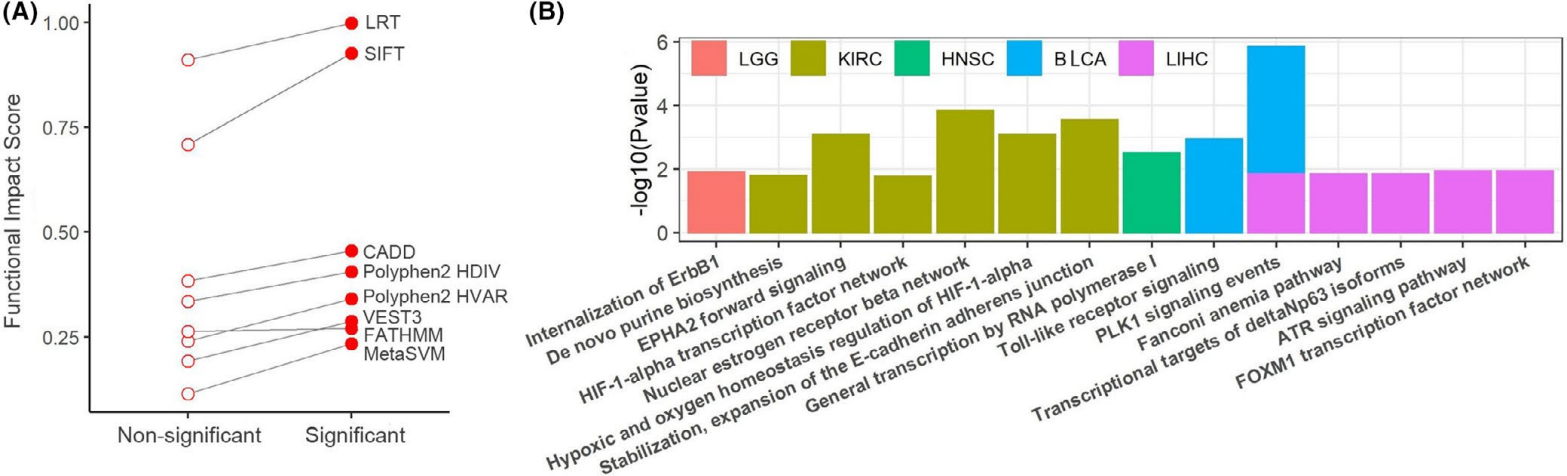
Functional characterization of prognostic gene‐proximal RNA‐editing sites (RESs). (A) Eight functional impact prediction scores were computed for RESs that were separated into two groups. The clinically relevant RESs (“Significant”) averaged higher functional impact scores than non‐clinically relevant RESs (“Non‐significant”). (B) Pathway enrichment analysis results using the host genes of prognostic RNA‐editing sites. PLK1 signaling events pathway has two colors because it was found enriched in two cancer types

By comparing the p‐value out of the ultimate survival analysis model (Equation 8), we assigned all gene‐proximal RESs into two groups: statistically non‐significant (adjusted *p*‐value ≥0.05) and statistically significant (adjusted *p*‐value <0.05). Each RES that was applicable to a mutation impact prediction algorithm was assessed with a predicted functional impact score, and the average functional impact scores of the two separate groups were compared. For all attempted prediction algorithms except FATHMM, the average functional impact score of the significant group was higher than that of the non‐significant group (Figure 5A). This result suggested that our refined prognostic RESs generally reside in more evolutionarily conserved genomic locations and their editing variations should lead to nontrivial functional impacts.

Using the 402 significant RESs’ host genes, we conducted pathway enrichment analysis by cancer and identified 15 significant pathways after adjusting for false discovery (Figure 5B). Many of the identified pathways have known associations with cancers. For example, *PLK1* signaling pathway, a key regulator of cell division, was significant in Bladder Urothelial Carcinoma (adjusted *p* < 0.0001) and liver hepatocellular carcinoma (adjusted *p* = 0.01). This pathway mediates estrogen receptor‐regulated gene transcription in breast cancer^55^ and it is associated with *TP53* inactivation, DNA repair deficiency in ER‐positive, Her2‐negative breast cancer.^56^ Another example is the *EPHA2* forward signaling pathway which was significant in KIRC (adjusted *p* = 0.0008). *EPHA2* expression has been shown positively associated with tumor size and Fuhrman nuclear grade in KIRC^57^ and promoting resistance to chemotherapy of sunitinib.^58^

Our initial RES data was previously analyzed by Han et al.^5^ who identified 1025 statistically significant survival RESs, 54 of which were also identified by us. The differences can be contributed to the different types of survival data (overall vs disease‐specific) and our more robust analysis strategy, where host gene expression and clinical covariates were accounted for. Furthermore, by resorting to several recent review articles,^14^, ^15^, ^59^, ^60^ there were 26 RESs with cancer‐related clinical significance that were covered by our dataset, which resided in 7 genes (*AZIN1*, *BLCAP*, *COG3*, *COPA*, *FLNB*, *GRIA2*, and *NEIL1*). In Table 4, we show the results from host gene correlation analysis (Equation 5) and three gradually refined Cox models (Equations 6, 7, and 8). Raw p‐values were used here instead of adjusted *p*‐values because we were focusing on a small list of predefined RESs. The editing level of 15 RESs had significant correlations with their host gene expression. One of these 15 RESs had significant prognostic values (*COG3* I635V in LUSC). Of the other 11 RESs, four had significant prognostic values after adjusting for host gene expression and clinical variables (*COG3* I635V in LUAD, *AZIN1* S367G in BRCA, *AZIN1* S367G in LUAD, and *BLCAP* Q5R in BLCA). This analysis demonstrates that RESs with true association with cancer prognosis can endure rigorous statistical adjustment for host gene expression and basic clinical variables.

**TABLE 4:** Revisiting clinically significant RNA editing sites that were individually implicated in cancers. *p*‐values less than 0.05 were bolded

Gene	Cancer type^§^	Residue change	Chromosome	Position	Raw *p*‐value			
					Equation (5)	Equation (6)	Equation (7)	Equation (8)
*NEIL1*	LUAD	K242R	15	75646087*	**3.86 × 10^−25^**	0.063	0.516	0.676
*NEIL1*	LUAD	K242R	15	75646086	**4.20 × 10^−25^**	0.070	0.825	0.708
*NEIL1*	LUSC	K242R	15	75646087*	**7.16 × 10^−13^**	0.775	0.989	0.921
*NEIL1*	LUSC	K242R	15	75646086	**1.11 × 10^−12^**	0.782	0.421	0.405
*COG3*	HNSC	I635V	13	46090371	**2.15 × 10^−12^**	0.050	0.072	0.321
*COG3*	KIRP	I635V	13	46090371	**3.48 × 10^−6^**	0.263	0.208	0.760
*COPA*	LIHC	I164V	1	160302244	**5.30 × 10^−6^**	0.093	0.083	0.206
*COG3*	BRCA	I635V	13	46090371	**6.14×10^−6^**	0.704	0.504	0.351
*COG3*	KIRC	I635V	13	46090371	**7.11 × 10^−6^**	0.075	0.087	0.114
*GRIA2*	GBM	R764G	4	158281294	**1.25 × 10^−4^**	0.398	0.411	0.448
*AZIN1*	CRC	S367G	8	103841636	**2.94 × 10^−4^**	0.712	0.773	0.969
*GRIA2*	LGG	R764G	4	158281294	**0.002**	0.869	0.159	0.822
*COG3*	LUSC	I635V	13	46090371	**0.003**	**0.028**	**0.024**	**0.007**
*BLCAP*	CRC	Q5R	20	36147563	**0.005**	0.877	0.547	0.551
*AZIN1*	LIHC	S367G	8	103841636	**0.011**	0.094	0.108	0.587
*BLCAP*	LGG	Q5R	20	36147563	0.060	0.240	0.329	0.061
*COG3*	LUAD	I635V	13	46090371	0.076	**0.024**	**0.014**	**0.016**
*BLCAP*	GBM	Q5R	20	36147563	0.160	0.966	0.968	0.838
*FLNB*	LIHC	M2269V	3	58141801	0.196	0.106	0.116	0.099
*BLCAP*	BLCA	Q5R	20	36147563	0.526	0.052	**0.024**	**0.010**
*COPA*	CRC	I164V	1	160302244	0.575	0.106	0.137	0.069
*BLCAP*	CESC	Q5R	20	36147563	0.612	0.593	0.528	0.522
*BLCAP*	LIHC	Q5R	20	36147563	0.912	0.618	0.953	0.844
*AZIN1*	BRCA	S367G	8	103841636	1.000	0.101	0.104	**0.015**
*AZIN1*	LUAD	S367G	8	103841636	1.000	**0.005**	**0.005**	**0.009**
*AZIN1*	LUSC	S367G	8	103841636	1.000	0.653	0.583	0.690

* In addition to the precisely matching genomic location of chr15:75646086 which certainly results in the K242R amino acid substitution, we also included the immediately adjacent A‐to‐G editing at chr15:75646087, which would also result in the K242R amino acid substitution if co‐occurring with the chr15:75646086 editing event.

§ Full cancer names are expanded in Table S1.

## 4 DISCUSSION

Previous studies touched upon RNA‐editing's implication in human diseases, but have not well elucidated RNA‐editing's involvement in cancer prognosis. In this study, we adopted a quantitative perspective to study RESs in 17 cancer types at single‐nucleotide resolution, addressing RES genomic distribution, RES‐gene correlation, RES survival association, and RES regulatory mechanism.

The foremost interesting finding is that the highest RNA‐editing level is observed in splicing junctions, in any cancer type or the pan‐cancer scope. Previous studies^61^, ^62^, ^63^ have shown that RNA editing can regulate alternative splicing, and ADAR‐regulated alternative splicing influences tumorigenesis.^64^ Our finding of the striking RNA‐editing level in splicing junctions strengthens the belief that RNA‐editing plays an important role in tumorigenesis‐relevant alternative splicing.

For the first time, we demonstrated that RNA‐editing level tends to be positively correlated with host gene expression. This finding may be intuitive, but it casts doubt on RES analysis results where host gene expression is not properly adjusted – association relations identified through unadjusted RNA‐editing level may merely be reflecting the latent effect of host gene expression. Thus, we recommend that any analysis that revolves around RNA‐editing level should consider adjusting for host gene expression. In our survival analyses in this work, we elected to adjust for host gene expression and other available demographic covariates. After multiple‐test correction, we identified 402 gene‐proximal RESs that were significantly associated with disease‐specific survival in 11 cancer types. An overwhelming majority of these 402 significant RESs were associated with poor survival (as opposed to good survival).

The current work consolidated RNA‐editing’s crucial involvement in cancers. This was grounded in multiple lines of evidence. First, we showed RNA‐editing level has prognostic value for hundreds of RESs in a wide range of cancers. Second, a majority of the prognostic RESs exert functional repercussion by altering RBP/miRNA binding sequences, and many targets of these RBPs/miRNAs had known cancer relevance. Thirdly, quite a few cancer‐related cellular pathways emerged in the functional enrichment analysis of prognostic RESs’ host genes. Lastly, our refined prognostic RESs generally reside in more evolutionarily conserved genomic locations than the other RESs that failed our rigorous survival model analysis.

Like the index of mutational burden, RNA‐editing number or level can be measured in aggregate as another sample‐level index. While early sporadic studies^12^, ^13^ reported decreased RNA editing level was associated with tumorigenesis or progression, a recent major study revealed that the increase in the total number of RNA‐editing events is correlated with poor prognosis.^16^ Here, globally speaking, we found that the increase in RNA‐editing level of individual RESs may predict an adverse cancer prognosis. We may conclude that an overall RNA‐editing burden can be built upon either the number of RESs or the average RNA‐editing level. More importantly, our work demonstrated that analysis of RNA‐editing level can be conducted at single‐nucleotide resolution with proper adjustment for basic clinical covariates, which offers room for discoveries of additional crucial biological mechanisms, such as altered cis‐regulatory elements linking to RBPs and miRNAs.

## CONFLICT OF INTEREST

None.

## AUTHOR CONTRIBUTIONS

YMW and YG wrote the manuscript; YMW, YG, and HY performed formal analysis; TG supervised the project.

## ETHICAL STATEMENT

All data used in this study are de‐identified public data. No ethical approval was a need from the institutional review board.

## Supporting information

Table S1

Table S2

Table S3

Table S4

Table S5

## Data Availability

All data used in this study were downloaded from Genomic Data Commons (https://gdc.cancer.gov/).
